# Prevention of Evisceration or Enucleation in Endogenous Bacterial Panophthalmitis with No Light Perception and Scleral Abscess

**DOI:** 10.1371/journal.pone.0169603

**Published:** 2017-01-05

**Authors:** Kuan-Jen Chen, Yen-Po Chen, An-Ning Chao, Nan-Kai Wang, Wei-Chi Wu, Chi-Chun Lai, Tun-Lu Chen

**Affiliations:** Department of Ophthalmology, Chang Gung Memorial Hospital, Chang Gung University College of Medicine, Tayouan, Taiwan; Albert-Ludwigs-Universitat Freiburg, GERMANY

## Abstract

Panophthalmitis is the most extensive ocular involvement in endophthalmitis with inflammation in periocular tissues. Severe inflammation of the anterior and posterior segments is frequently accompanied by corneal opacity, scleral abscess, and perforation or rupture. Enucleation or evisceration was the only remaining viable treatment option when all options to salvage the eye had been exhausted. The purpose of this retrospective study is to examine the outcomes of patients with endogenous bacterial panophthalmitis, no light perception and scleral abscess who were treated with multiple intravitreal and periocular injections of antibiotics and dexamethasone. Evaluation included spreading of infection to contiguous or remote sites, following evisceration or enucleation, and sympathetic ophthalmia. Eighteen patients were diagnosed with EBP, with liver abscesses in eight patients, retroperitoneal infection in four, pneumonia in two, infective endocarditis in one, cellulitis in one, drug abuse in one, and mycotic pseudoaneurysm in one. Culture results were positive for *Klebsiella pneumoniae* in 12 patients, *Streptococcus spp*. in three, *Pseudomonas aeruginosa* in one, *Escherichia coli* in one, and *Staphylococcus aureus* in one. The average number of periocular injections was 2.2, and the average number of intravitreal injections was 5.8. No eye required evisceration or enucleation and developed the spreading of infection to contiguous or remote sites during the follow-up. No sympathetic ophthalmia was observed in the fellow eye of all patients. Prevention of evisceration or enucleation in patients with EBP, NLP and scleral abscess can be achieved by multiple intravitreal and periocular injections of antibiotics and dexamethasone.

## Introduction

Panophthalmitis is the most extensive ocular involvement in endophthalmitis with inflammation in periocular tissues. Severe inflammation of the anterior and posterior segments is frequently accompanied by corneal opacity, scleral abscess, and perforation or rupture. The disease develops rapidly and the prognosis is poor. The management of patients with intractable panophthalmitis remains controversial. In previous reports, enucleation or evisceration was the only remaining viable treatment option when all options to salvage the eye had been exhausted [1–8]. Because of a relatively high extrusion rate and the potential for sympathetic ophthalmia in the other eye, enucleation had been recommended [2,9,10]. In recent decades, evisceration with either delayed or immediate implant placement has been recommended in patients with endophthalmitis [5–8]. Although delayed primary closure has certain theoretical advantages, primary implantation of orbital implants can prevent prolonged hospitalization and the need for additional surgeries.^5-8^ However, attempts at implant placement with evisceration are typically abandoned during surgery because the implant is considered unlikely to remain within the necrotic sclera [6]. Melting of the scleral necrotic tissue causes a high extrusion rate of the orbital implant.

We addressed a new treatment to prevent evisceration and enucleation for intractable panophthalmitis in letters and correspondence [11–12]. The purpose of this retrospective study is to examine the outcomes of patients with endogenous bacterial panophthalmitis, no light perception and scleral abscess who were treated with multiple intravitreal and periocular injections of antibiotics and dexamethasone.

## Materials and Methods

The study protocol was approved by the Institutional Review Board of Chang Gung Memorial Hospital, Taiyuan, Taiwan, and adhered to the tenets of the Declaration of Helsinki and all applicable regulatory and legal requirements. The study was a retrospective case series. We enrolled patients who developed endogenous bacterial panophthalmitis with no light perception at one tertiary referred center (Chang Gung Memorial Hospital, Taoyuan, Taiwan) from May 2005 to December 2014. Patient records were de-identified and analyzed anonymously. The inclusion criteria were as follows: (1) A primary infectious source was identified in patients with endogenous endophthalmitis. The isolates from patient’s body fluids were cultured with growth of bacteria. (2) The eye with endogenous endophthalmitis progressed to panophthalmitis with scleral abscess. (3) The vision of eye involved was no light perception. (4) The patients were followed up for at least 12 months. (5) The eye involved met the criteria of evisceration or enucleation. All patients were recommended for enucleation or evisceration with or without an orbital implant by oculoplastic surgeons, and were referred for a second opinion with no need of evisceration or enucleation. The exclusion criteria were as follows: (1) Panophthalmitis was caused by surgery or trauma. (2) The organisms cultured were fungi or no organism was isolated. (3) The primary infectious source was not identified. (4) The interval of onset between primary infectious sources identified and endophthalmitis was more than 14 days. The demographic data at presentation, management, complication, and cosmetic outcome were reviewed. The main treatment for endogenous bacterial panophthalmitis included intravitreal and periocular injections of antibiotic/dexamethasone combinations. The periocular treatment included subconjunctival and sub-Tenon’s injections. Descriptive statistics were used for data analysis.

### Dosage and concentration of antibiotics and dexamethasone

Before the culture results were obtained, the first intravitreal injections included vancomycin for gram-positive organisms, ceftazidime for gram-negative organisms, and dexamethasone. Ceftazidime (500 mg/vial) or vancomycin (500 mg/vial) was diluted with 5 mL of normal saline (0.9% NaCl), and the concentration of ceftazidime and vancomycin was 100 mg/mL (10 mg/0.1mL). The preservative-free dexamethasone (5 mg/mL) was mixed with diluted vancomycin or ceftazidime at a ratio of 1:1.

### Procedures of periocular and intravitreal injections

All procedures were performed under topical anesthesia. Periocular injections of antibiotic/dexamethasone combinations were administered around the involved quadrants with scleral abscesses. In patients with corneal ring-shaped infiltration, slower injections of intravitreal agents were administrated to prevent corneal laceration caused by high intraocular pressure. Eyes with corneal laceration were slowly intravitreally injected until the injected fluid was identified from corneal laceration without prolapse of intraocular contents. The volume of intravitreal injection was 0.1 to 0.3 mL in eyes without corneal laceration and 0.3 to 0.5 mL in eyes with corneal laceration. According to the location of scleral abscesses, the volume of periocular injection was in total 0.3 mL to 1.0 mL for one to four quadrants. The frequency of injection depended on the resolution of panophthalmitis. After the scleral abscess subsided, repeated intravitreal injections of antibiotic/dexamethasone combinations were performed. The interval of injection began between 48 and 72 hours for 1 to 2 weeks, followed by twice a week, and then once a week until the infection and inflammation were under control.

### Evaluation of complications

The patients were followed up to evaluate the following complications: resolution of infection and inflammation, laceration of corneal and scleral necrosis, and spreading of infection to contiguous or remote sites. They were also evaluated following evisceration or enucleation, sympathetic ophthalmia, and successful fitting of the prosthesis.

## Results

**Fig 1:**
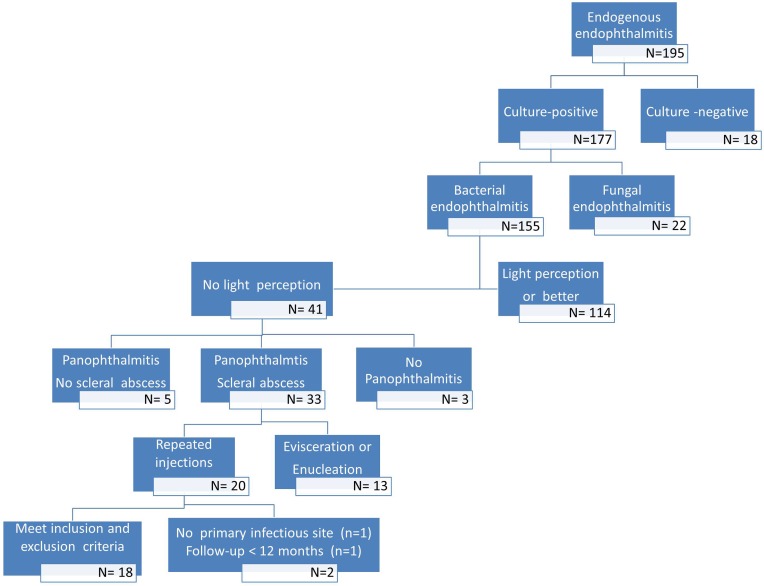
Flow of participants into the study.

There were 195 patients with presumed endogenous endophthalmitis in this study period. No organism was isolated in 18 patients and fungal infection was identified in 22 patients. The primary infectious source was not identified in 17 patients. Three patients were excluded because the interval of onset between primary infectious sources identified and endophthalmitis was more than 14 days. Finally, thirty-three eyes (patients) developed bacterial panophthalmitis, scleral abscess and no light perception. Among them, eighteen patients met the inclusion and exclusion criteria. Thirteen patients received evisceration or enucleation. The number of patients with presumed endogenous endophthalmitis is shown in Fig 1.

A total of 18 patients with bacterial panophthalmitis, scleral abscess and no light perception were evaluated (Table 1). Thirteen right eyes and five left eyes were involved. The age ranged from 39 to 84 years (mean, 64±15 years; median, 67 years), and there were 10 male patients and 8 female patients. The mean follow-up duration was 39 months (range, 12–96 months).

**Table 1:** Demographic data in patients with bacterial panophthalmitis and scleral abscess.

Patient	Gender/			Cultures	Corneal	Systemic	Primary	Secondary	No. of	No. of
No.	Age/	Cause	Organism	B/P/E	ring-shaped	antibiotics	antibiotics	antibiotics	periocular	intravitreal
	Eye				infiltration				injections	injections
1	M/84/OD	Pneumonia	*Pseudomonas aeruginosa*	+/NA/+	-	CAZ	CAZ	CAZ	2	5
2	M/84/OD	Liver abscess	*Klebsiella pneumoniae*	+/+/+	-	CRO	CAZ	CAZ	2	6
3	F/45/OD	Pyelonephritis	*Escherichia coli*	-/+/+	+	CRO	CAZ	CAZ	2	6
4	M/46/OS	Liver abscess	*Klebsiella pneumoniae*	+/NA/+	-	CRO	CAZ	CAZ	2	6
5	F/66/OS	Liver abscess	*Klebsiella pneumoniae*	+/+/+	-	CRO	CAZ	CAZ	3	10
6	M/84/OD	Mycotic	*Streptococcus agalactiae*	+/-/+	-	PCN	VAN+CAZ	VAN	2	4
		pseudoaneurysm								
7	F/48/OD	Cellulitis	*Staphylococcus aureus*	+/+/+	-	OXA	VAN +CAZ	VAN	2	5
8	F/53/OD	Liver abscess	*Klebsiella pneumoniae*	+/NA/+	-	CRO	CAZ	CAZ	2	5
9	M/70/OD	Liver abscess	*Klebsiella pneumoniae*	+/+/-	+	CRO	CAZ	CAZ	3	7
10	F/50/OD	Renal abscess	*Klebsiella pneumoniae*	+/+/+	-	CRO	CAZ	CAZ	2	4
11	M/67/OS	Retroperitoneal	*Klebsiella pneumoniae*	+/+/+	-	CRO	CAZ	CAZ	2	5
		(nonrenal) abscess								
12	M/39/OS	Drug abuse	*Klebsiella pneumoniae*	+/+/+	+	CRO	VAN+CAZ	CAZ	2	4
13	M/58/OD	Liver abscess	*Klebsiella pneumoniae*	-/+/+	-	CRO	CAZ	CAZ	3	10
14	F/51/OD	Pyelonephritis	*Klebsiella pneumoniae*	+/+/-	-	CRO	CAZ	CAZ	2	5
15	F/78/OD	Liver abscess	*Klebsiella pneumoniae*	+/-/-	+	CRO	CAZ	CAZ	2	6
16	M/70/OD	Liver abscess	*Klebsiella pneumoniae*	+/+/-	+	CRO	CAZ	CAZ	3	8
17	F/84/OS	Pneumonia	*Streptococcus agalactiae*	+/NA/-	-	CRO	VAN+CAZ	VAN	2	5
18	M/72/OD	Infective	*Streptococcus pneumoniae*	+/NA/+	-	VAN+CRO	VAN+CAZ	VAN	2	4
		endocarditis								

B, blood; CAZ, ceftazidime; CRO, ceftriaxone; E: eye; NA, not available; OXA, Oxacillin; P, primary infectious source; PCN, penicillin; VAN, vancomycin

Eighteen patients were diagnosed as endogenous endophthalmitis, with liver abscesses in eight patients, renal infection in three, pneumonia in two, infective endocarditis in one, cellulitis in one, retroperitoneal (nonrenal) abscess in one, drug abuse in one, and mycotic pseudoaneurysm in one. Eighteen isolates from body fluids of 18 patients were cultured. Culture results were positive for *Klebsiella pneumoniae* in 12 patients, *Streptococcus spp*. in three patients, *Pseudomonas aeruginosa* in one patient, *Escherichia coli* in one patient, and *Staphylococcus aureus* in one patient. The three *Streptococcus* species included *Streptococcus agalactiae* in one patient, *Streptococcus pyogenes* in one patient, and *Streptococcus pneumoniae* in one patient. All gram-positive isolates were susceptible to vancomycin, and all gram-negative isolates were susceptible to ceftazidime. Associated clinical findings at the time of referral were scleral abscess in 13 patients (72%) and scleral abscess with corneal ring-shaped infiltration in five patients (28%). Before inclusion in this study, two eyes with corneal ring-shaped infiltration developed corneal laceration after a previous intravitreal injection of antibiotics.

No eyes developed extrusion of intraocular contents during the intravitreal and periocular injections. The number of periocular injections was two or three, and the average number of injections was 2.2. The number of intravitreal injections was three to ten, and the average number of injections was 5.8. All eyes except one showed resolution of infection and inflammation within 10 days to 1 month. One patient with *Klebsiella* liver abscess developed recurrent endophthalmitis after stabilization for 1 month. After another injection of intravitreal ceftazidime and dexamethasone, no recurrence occurred during the 24-month follow-up. Patients with endogenous infectious sources were treated with systemic antibiotics to control the primary infectious source. Before the organisms and antibiotic susceptibility testing were identified, patients were treated with the empiric systemic antibiotics. The choice of systemic antibiotics depended on the antibiotic susceptibility testing of organisms isolated from tissue samples including blood, eye, and/or primary infectious source.

No eye required evisceration or enucleation and developed the spreading of infection to contiguous or remote sites during the follow-up. No sympathetic ophthalmia was observed in the fellow eye of all patients during the follow-up. All eyes had successful fitting of the prosthesis and resulted in a favorable quality of life; however, two eyes with corneal laceration developed obvious phthisis bulbi with the need of a larger and thicker ocular prosthesis.

## Discussion

Panophthalmitis is a severe type of endophthalmitis. Panophthalmitis is an acute inflammation of the eyeball involving all its structures and extending to the orbit, and is typically caused by virulent pyogenic organisms. The infection can result from a penetrating injury to the eye, ocular surgery, or septicemia, or can spread from a pus-producing infection in another part of the body. When medical and surgical options to salvage the eye are exhausted in a patient with intractable panophthalmitis, the only option that remains is to remove the eye through evisceration or enucleation, according to a literature review [1–8]. An increasing number of surgeons choose to perform evisceration because of its superior long-term cosmetic outcome and faster recovery compared with enucleation [5–8]. However, attempts at implant placement are typically abandoned during surgery because the implant is likely to erode through necrotic sclera [6]. The integrity of the necrotic conjunctiva is typically friable and difficult to suture with frequent cheese wiring. The necrotic sclera, conjunctiva, and Tenon’s capsule cannot protect an implant if the surgeon chooses evisceration. Evisceration with a primary implant causes a high extrusion rate of the orbital implant in such cases. Furthermore, patients with panophthalmitis typically hope to preserve the eye globe and experience a psychological impact from the loss of the eyeball. Globe salvage, defined as the avoidance of enucleation or evisceration, can be achieved in such patients undergoing the treatment in our study.

The panophthalmitis in our patients was generally more severe than that in the patients of previous reports [5–8]. We enrolled only patients who developed panophthalmitis with scleral abscess with or without corneal ring-shaped infiltration. All patients developed endogenous endophthalmitis caused by virulent organisms. Endogenous bacterial endophthalmitis typically metastasizes to the choroid and develops choroidal abscesses. With delayed diagnosis and without prompt and frequent treatment, endophthalmitis may progress to panophthalmitis with the development of scleral abscesses [3]. Moreover, the cornea develops ring-shaped infiltration and necrotic laceration. In this situation, enucleation or evisceration without an immediate orbital implant has been recommended in previous reports [1,6].

Although purulent ocular infection is believed to destroy the uveal tissue and antigens to an extent that such eyes do not induce sympathetic ophthalmia, fear of sympathetic ophthalmia is a classic concern whenever evisceration is performed [9,10]. Few case reports have described the presence of endophthalmitis in eyes with sympathetic ophthalmia [13–15]. Sympathetic ophthalmia also has been reported after evisceration for endophthalmitis [9]. Sympathetic ophthalmia could be triggered by postsurgical endophthalmitis or the evisceration procedure in such cases. In the current case series, there was no occurrence of sympathetic ophthalmia during at least one-year follow-up. However, an enormous sample size and longer duration of follow-up would be needed to detect an occurrence after such treatment for panophthalmitis.

In this case series, eyes treated with antibiotic/dexamethasone combinations showed an improved result and a quick resolution of panophthalmitis without the need of enucleation or evisceration. With the use of antibiotics, dexamethasone can substantially reduce inflammation quickly without the deterioration or spreading of infection. Although vancomycin equally mixed with dexamethasone mildly develops precipitation, ceftazidime can be equally mixed with dexamethasone without developing precipitation. A mixture of ceftazidime and vancomycin also can result in clinically visible precipitation, and the formation of a precipitate associated with intravitreal injections was correlated with the diffusion rate of the antibiotics. Precipitation increased at lower temperatures, lower media volumes, and higher antibiotic concentrations [16]. However, the precipitate and the supernatant retains substantial antibacterial activity, thus confirming the efficacy of combination therapy with vancomycin and ceftazidime in the management of bacterial endophthalmitis [17,18].

All patients were successfully fitted with an ocular prosthesis. Sixteen eyes without corneal laceration had a superior cosmetic result because there was no substantial loss of intraocular contents during the treatment. Two eyes with corneal laceration did not maintain the intraocular pressure during the treatment and developed obvious phthisis bulbi with the need of a thicker and larger ocular prosthesis. Therefore, evisceration and immediate implant placement may be optional in such cases after panophthalmitis is under control. The current treatment is substantially less expensive, has less psychological trauma, and requires only simple management without surgery. The disadvantages are that it requires multiple injections and causes obvious phthisis bulbi in eyes with corneal laceration. Multiple injections of an ultrahigh dose of intravitreal antibiotic/dexamethasone combinations could preserve the globe without enucleation or evisceration in the current study. Although the globe eventually showed phthisis bulbi, the patients experienced less psychological problems and required fewer subsequent surgeries for better cosmetic results. The limitations of this study included a short follow-up (one year) for some patients, the absence of a control group (without steroid treatment), and the absence of other types of endophthalmitis such as traumatic or postoperative endophthalmitis.

In conclusion, our rationale in this retrospective study was to investigate the prevention effect of evisceration or enucleation by using multiple intravitreal and periocular injections of high-dose antibiotic/dexamethasone combinations in patients with endogenous bacterial panophthalmitis, no light perception and scleral abscess. We demonstrated that severe panophthalmitis does not persist in the sclera, which is retained in the orbit, or surrounding tissues. Results also showed that the treatment could be tolerated easily without the progression of infection. This study should form a basis for the future examination of this topic by large, multicenter, randomized, prospective studies. The primary and secondary outcomes of such studies should include the resolution of infection, long-term risk of sympathetic ophthalmia, and an objective assessment of the quality of life after treatment.
